# The effect of tibialis posterior tendon transfer on anatomical foot parameters and functional outcomes in drop foot patients: a single center study

**DOI:** 10.1007/s00402-025-06179-y

**Published:** 2026-02-02

**Authors:** Umut Can Duvarci, Sefa Erdem Karapinar, Recep Dincer, Serdar Kamil Cepni, Yakup Barbaros Baykal, Vecihi Kirdemir

**Affiliations:** 1 Sandıklı state hospital, Afyonkarahisar, Turkey; 2 Suleyman Demirel University, Isparta, Turkey; 3 Umraniye Training and Research Hospital, Istanbul, Turkey

**Keywords:** Foot drop, Tibialis posterior tendon transfer, Interosseous membrane, Functional outcomes, Radiological parameters.

## Abstract

**Purpose:**

This retrospective single-center study aimed to evaluate the anatomical and functional outcomes of tibialis posterior tendon transfer in patients with foot drop. Foot drop is characterized by weakness or loss of ankle dorsiflexion, eversion, and toe extension, commonly resulting from neurological, systemic, or traumatic causes. Tibialis posterior tendon transfer improves ankle dorsiflexion function rather than restoring normal ankle function, and it also eliminates deforming forces on the medial foot. This study aimed to evaluate the anatomical and functional outcomes of tibialis posterior tendon transfer in patients with foot drop.

**Methods:**

This single-center retrospective study included 20 patients who underwent tibialis posterior tendon transfer via the interosseous membrane between 2016 and 2023. Radiological evaluation included assessment of the tibiotalar angle and hindfoot alignment. Evaluation of the medial longitudinal arch (MLA) was based on measurements of the calcaneus–first metatarsal angle, lateral talus–first metatarsal angle (Meary’s angle), lateral talocalcaneal angle, talohorizontal angle, calcaneal pitch angle, talonavicular coverage angle, anteroposterior (AP) talocalcaneal angle, and AP talus–first metatarsal angle. Surgical success and functional recovery were assessed using the Stanmore score and the criteria described by Carayon et al. The primary endpoint was the postoperative improvement in active dorsiflexion and medial longitudinal arch parameters. The Carayon criteria evaluate active dorsiflexion (DF), active plantarflexion (PF), and active range of motion (ROM). The Stanmore score assesses foot position, active DF, muscle strength grade, functional activities, ability to use normal footwear, orthosis use, and pain.

**Results:**

The mean age was 38.25 years (range, 22–80). The mean duration of paralysis was 45.55 months, and the mean follow-up period was 42.6 months. According to the Stanmore score, outcomes were excellent in 30% of patients, good in 30%, fair in 20%, and poor in 20%. The mean postoperative active dorsiflexion gain was 9° (SD ± 5.6). Changes in the lateral talocalcaneal, lateral talus–first metatarsal, and talohorizontal angles showed a significant relationship with the duration of paralysis, indicating a tendency toward MLA collapse; however, no patients developed pes planus or other deformities.

**Conclusion:**

Tibialis posterior tendon transfer effectively reduces the need for orthotic devices and achieves satisfactory restoration of active ankle dorsiflexion in patients with foot drop. Careful tension adjustment and proper positioning during surgery are essential for optimal alignment and functional recovery.

## Introduction

Foot drop is characterized by weakness or loss of ankle dorsiflexion, eversion, and toe extension, which may result from neurological, systemic, or traumatic causes. Patients typically exhibit a high-stepping gait pattern to compensate for the inability to clear the foot during the swing phase of gait, leading to increased energy expenditure and abnormal gait biomechanics [1, 2]. In advanced cases, pelvic tilt may develop over time, and equinovarus deformity may occur due to the pull of the tibialis posterior tendon.

The primary goal in the treatment of foot drop is to improve dorsiflexion strength and achieve a functional gait pattern, as complete restoration of normal gait mechanics is not expected after tibialis posterior tendon transfer. Ankle-foot orthoses (AFOs) that restrict excessive plantarflexion beyond the neutral position may be used to facilitate ambulation. Surgical options for foot drop include tenodesis, arthrodesis, and tendon transfer procedures. Given that the tibialis posterior muscle is the main dynamic stabilizer of the medial longitudinal arch, its transfer may theoretically lead to acquired flatfoot deformity [3].

In most cases, foot drop results from traumatic injuries involving the common peroneal or sciatic nerves. Such patients often undergo primary procedures including nerve repair, nerve grafting, or nerve transfer. These patients are typical candidates for tibialis posterior tendon transfer [4]. Successful tendon transfer requires a flexible donor muscle, a straight line of pull, and a supple recipient joint. Tendon transfer is contraindicated in rigid equinus deformity that cannot be passively corrected [5].

This study aimed to evaluate both the functional and radiological outcomes in patients who underwent tibialis posterior tendon transfer via the interosseous membrane for the treatment of foot drop. We hypothesized that tibialis posterior tendon transfer would improve functional dorsiflexion while maintaining medial longitudinal arch alignment. This study was designed as a retrospective single-center analysis.

## Materials and methods

### Patient selection

Between 2016 and 2023, patients who underwent tibialis posterior tendon transfer for foot drop in the Orthopedics and Traumatology Department were retrospectively reviewed. The study received ethical approval from the Institutional Review Board (Approval No: 363, dated 29.12.2023). Written informed consent was obtained from all patients.

Inclusion criteria were: age ≥ 18 years, minimum follow-up of 12 months, and tibialis posterior tendon transfer performed via the interosseous membrane. Of 28 patients initially identified, 20 patients met the inclusion criteria. Eight patients were excluded due to lack of preoperative radiographs or incomplete hospital records.

### Patient demographics

- Mean age: 38.25 years (range, 20–80 years).
- Mean duration of paralysis: 45.5 months (range, 2–252 months).
- Mean follow-up period: 42.6 months (range, 12–85 months).
- Side affected: right foot (*n* = 11), left foot (*n* = 9).
- Gender: male (*n* = 11), female (*n* = 9).

Etiologies of foot drop included acetabular fracture (*n* = 3), peroneal nerve injury (*n* = 3), iatrogenic causes (*n* = 3), complications following lumbar disc herniation surgery (*n* = 2), tumor compression (*n* = 2), gunshot injury (*n* = 2), total hip arthroplasty (*n* = 1), pressure injury after circular cast (*n* = 1), crush injury due to earthquake (*n* = 1), idiopathic causes (*n* = 1), and cerebral palsy sequelae (*n* = 1).

### Surgical technique

**Fig. 1:**
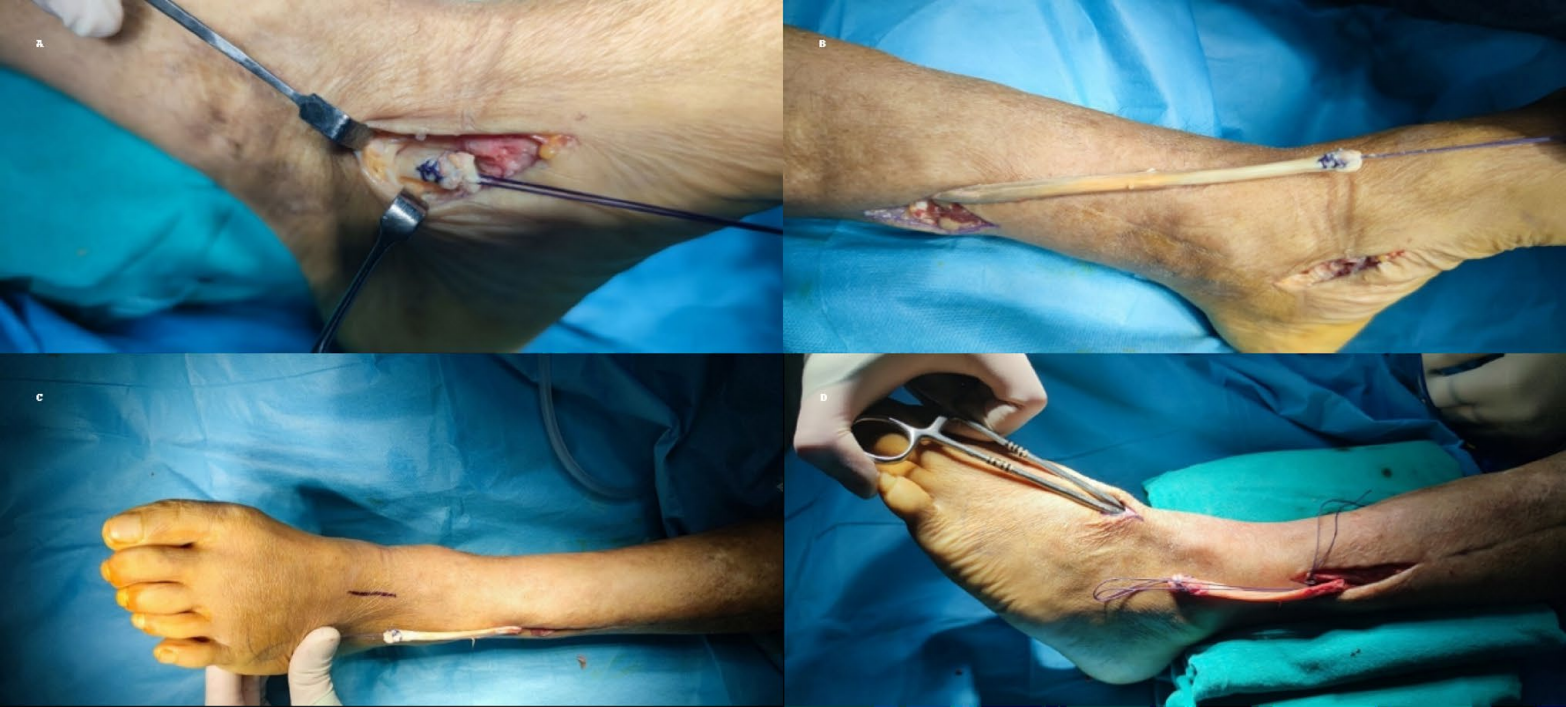
Surgical stages of tibialis posterior tendon transfer. (**A**. Placing a marker suture on the tendon. **B**. Proximally removing the tendon. **C**. Transfer of the tendon from the medial side to the lateral side. **D**. Sending the curved clamp under the extensor retinaculum from the dorsal aspect of the foot to the proximal lateral incision.)

A longitudinal incision (3–4 cm) was made over the navicular bone on the medial side of the foot to identify and detach the tibialis posterior tendon from its insertion. The distal end of the tendon was tapered and marked using a Krackow suture technique with 0 Vicryl suture to facilitate passage through the bone tunnel in the lateral cuneiform. A second incision (~ 5 cm), was made proximal to the medial malleolus to expose the musculotendinous junction of the tibialis posterior tendon. The neurovascular structures surrounding the muscle belly were protected, and the tendon was mobilized proximally. A third longitudinal incision (2 cm), was made laterally over the distal tibiofibular syndesmosis, keeping anterior to the fibula. The interosseous membrane was carefully opened, and a window approximately 3–4 cm wide was created. A clamp was passed through the interosseous membrane from posterior-medial to anterior-lateral, and the tendon was pulled from the medial to the lateral side of the leg, staying posterior to the tibia. After exiting the interosseous membrane, the tendon passes anterior to the fibula and deep to the extensor digitorum longus muscle belly, then continues beneath the extensor retinaculum toward the dorsal incision over the lateral cuneiform. Under fluoroscopic guidance, the center of the lateral cuneiform was located, and a fourth longitudinal incision (3 cm) was made dorsally. The extensor tendons and superficial peroneal nerve were preserved. A tunnel was drilled through the lateral cuneiform for tendon passage. A soft tissue tunnel was prepared under the extensor retinaculum to bring the tendon to the dorsal foot (Fig. 1).

**Fig. 2:**
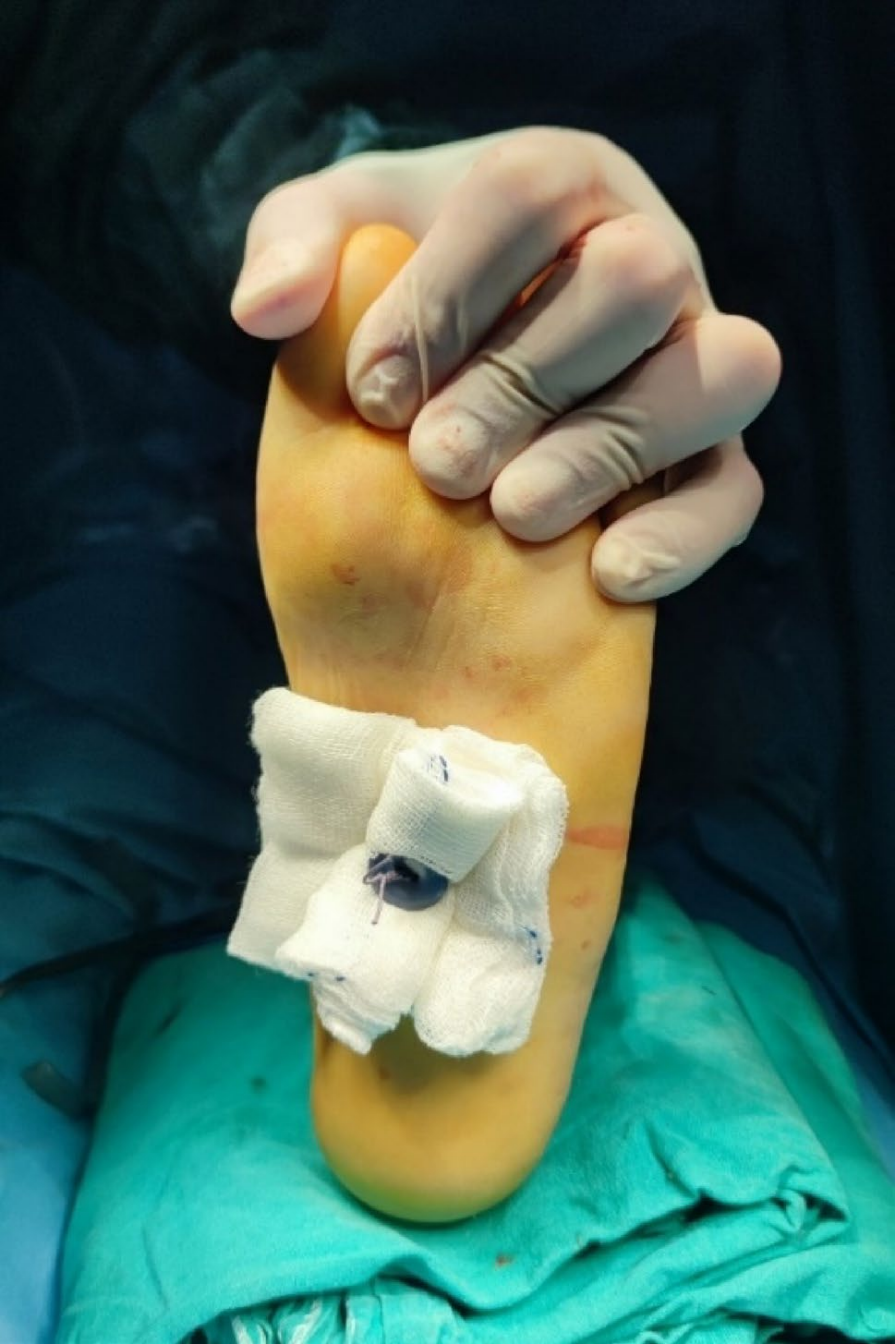
Passing the marker sutures to the button and tying it tightly by adjusting the foot tension. In order to prevent necrosis due to the compression of the button on the plantar surface, the K wire is passed through the sponge after leaving the skin and the button is supported with a sponge under the button

A Kirschner wire was passed through the bone tunnel, exiting the plantar aspect of the medial arch. The marking sutures were passed through the wire tract, and the tendon was tensioned with the ankle held in 10° dorsiflexion. The sutures were tied securely over a button placed on the plantar surface, with a sponge pad to prevent skin necrosis (Fig. 2). Additional fixation was provided with sutures to the periosteum at the tunnel entrance. The incisions were closed, and a short leg circular cast was applied with the ankle in dorsiflexion.

The button is removed after 6 weeks when the cırcular cast ıs removed.

### Postoperative rehabilitation and follow-up

Postoperatively, patients were immobilized in a short leg circular cast for six weeks. After cast removal, an ankle-foot orthosis (AFO) was applied to prevent plantarflexion. Active dorsiflexion exercises and re-education of the transferred muscle were initiated. Full weight-bearing was permitted at three months postoperatively.

### Radiographic evaluation

During the evaluation of the ankle, the anatomical structures and functional characteristics of the distal tibia, talus, and calcaneus must be considered. All radiographs were obtained in a standardized weight-bearing standing position. Additionally, the alignment of these structures in the sagittal and coronal planes should be taken into account. Radiological evaluation includes assessment of the tibiotalar angle and hindfoot alignment. For the medial longitudinal arch (MLA), the following measurements are used: calcaneus–first metatarsal angle, lateral talus–first metatarsal angle (Meary’s angle), lateral talocalcaneal angle, talohorizontal angle, calcaneal pitch angle, talonavicular coverage angle, anteroposterior (AP) talocalcaneal angle, and AP talus first metatarsal angle.

*Tibiotalar angle:* In normal alignment, the mechanical and anatomical axes of the tibia are considered to be the same. In the AP plane, these axes pass through the midpoint between the malleoli, the center of the ankle joint, or the midpoint of the tibiotalar articular surface, forming a 90° angle with the distal articular surface of the tibia. In a normally aligned ankle, there is no angulation between the dome of the talus and the distal tibial articular surface. In the sagittal plane, the mid-diaphyseal mechanical axis of the distal tibia passes through the lateral process of the talus, which serves as the center of rotation for the ankle joint. The distal tibial articular orientation line forms a 10° angle with the ground in the sagittal plane and an 80° angle with the anatomical axis of the tibia.

**Fig. 3:**
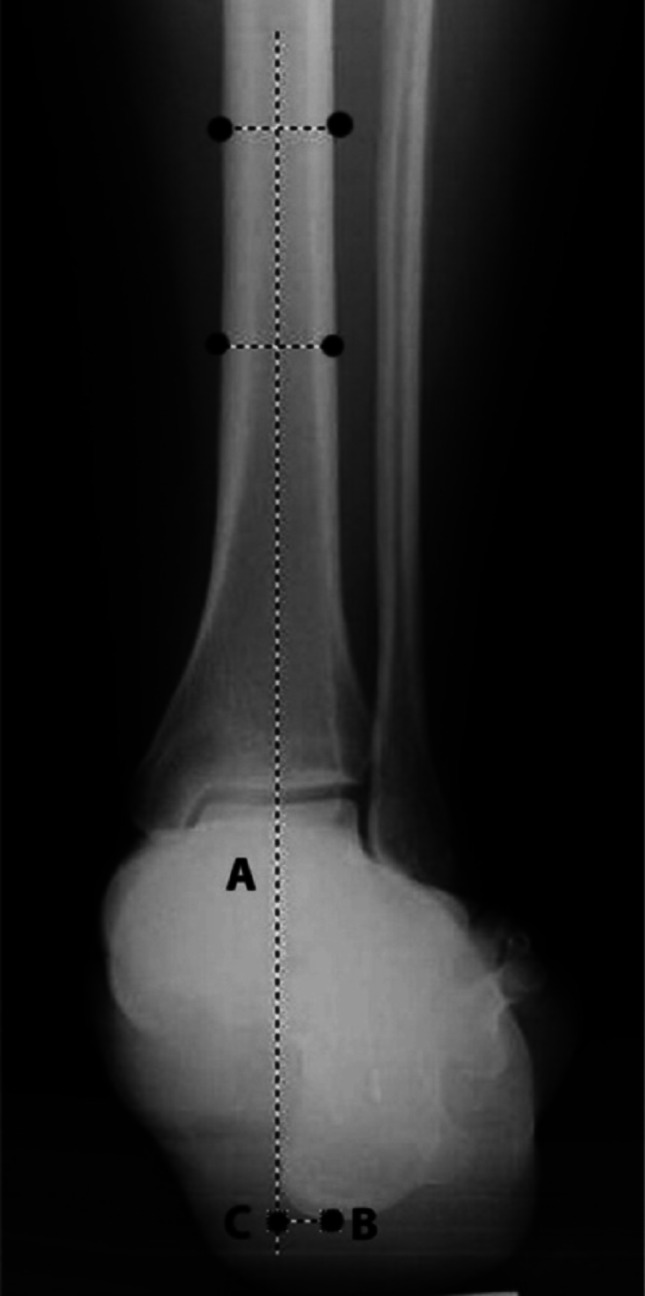
Measurement technique for hindfoot alignment in the coronal plane (visible moment arm). The A line is defined by bisecting the tibia at a point 10–15 cm proximal to the medial tibial plafond. Point B is identified as the lowest point of the calcaneus. The visible moment arm is determined by measuring the perpendicular distance between the A line and point B (BC line). If the weight-bearing axis of the leg (A line) is medial to point B, this indicates a positive value (valgus calcaneus); if the A line is lateral to point B, the value is negative (varus calcaneus)^*2*^

**Fig. 4:**
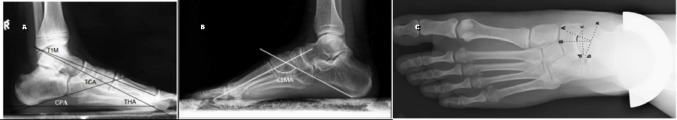
Measurement technique for hindfoot alignment in the coronal plane (visible moment arm). The A line is defined by bisecting the tibia at a point 10–15 cm proximal to the medial tibial plafond. Point B is identified as the lowest point of the calcaneus. The visible moment arm is determined by measuring the perpendicular distance between the A line and point B (BC line). If the weight-bearing axis of the leg (A line) is medial to point B, this indicates a positive value (valgus calcaneus); if the A line is lateral to point B, the value is negative (varus calcaneus) [6]

*Measurement of hindfoot alignment:* Hindfoot alignment was evaluated using weight-bearing Saltzman hindfoot alignment views. Hindfoot alignment is assessed on AP radiographs by drawing a line bisecting the tibia 10–15 cm proximal to the tibial plafond and marking the lowest point of the calcaneus. The perpendicular distance indicates the varus or valgus position of the hindfoot (Fig. 3, Fig. 4) [6].

**Fig. 5:**
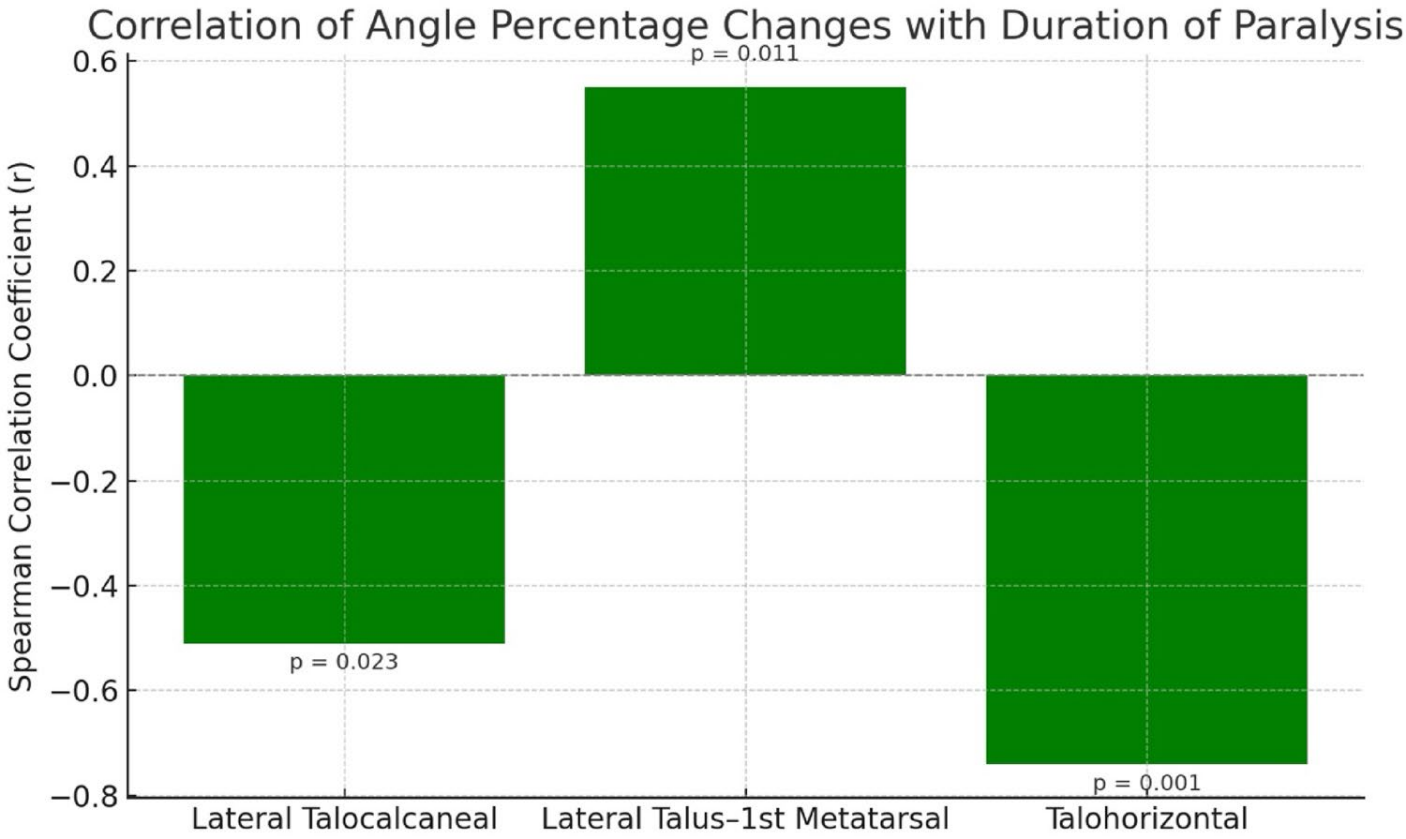
Representation of measured angles. (**A**. Angles measured on lateral radiograph. THA: Talohorizontal Angle; CPA: Calcaneal Pitch Angle; T1M: Talus-1st Metatarsal Angle; TCA: Talocalcaneal Angle. **B**. The calcaneus-first metatarsal angle is the angle measured between the undersurface of the calcaneus and the axis of the first metatarsal (C1MA: calcaneus-1st metatarsal angle) **C**. Technique for measuring the AP talonavicular overlap angle. Points a, b = medial and lateral edges of the articular surface of the talus (respectively). Points c, d = medial and lateral edges of the navicular articular surface (respectively). Line A is perpendicular to ab and line B is perpendicular to cd. The AP talonavicular coverage angle is the angle formed by the intersection of lines A and B^2^.)

The success of surgery and functional recovery were evaluated using the Stanmore scale and the criteria defined by Carayon et al. The Stanmore score assesses foot position, active dorsiflexion, muscle strength, gait, footwear compatibility, orthosis use, and pain on a 100-point scale. The Carayon criteria evaluate active dorsiflexion, plantarflexion, and overall ankle range of motion. Carayon et al. criteria are evaluated according to active dorsiflexion (DF), active plantarflexion (PF) and active range of motion (ROM). In the Stanmore scale; foot position, active DF, degree of muscle strength, function, normal footwear use, orthotic use and pain are evaluated on a 100-point scale (Fig.5) [7].

### Statistical analysis

All statistical analyses were performed using IBM SPSS Statistics version 25.0 (IBM Corp., Armonk, NY, USA). Continuous variables were presented as mean ± standard deviation (SD) or median (range). Categorical variables were expressed as frequencies and percentages. The chi-square test was used for categorical variables. The Kolmogorov–Smirnov and Shapiro–Wilk tests assessed the normality of continuous data, and the Levene test checked for homogeneity of variances. For non-normally distributed continuous variables, the Mann–Whitney U, Kruskal–Wallis H, and independent sample median tests were used. Bonferroni correction was applied for multiple comparisons. Spearman’s rho test was used for correlation analysis. A p-value < 0.05 was considered statistically significant. Due to the retrospective design, an a priori power analysis was not performed.

## Results

The mean active dorsiflexion (DF) gain was 9° (SD ± 5.6). No statistically significant correlation was found between DF gain and duration of paralysis or age (*p* > 0.05)(Table 1,Table 2).

**Table 1:** Relationship between dorsiflexion gain and duration of paralysis/age. There was no significant difference in mean DF gain based on gender (*p* > 0.05):

		DF Gain
Duration of Paralysis (months)	**r**	0.040
	**p**	0.868
Age	**r**	0.063
	**p**	0.793

**Table 2:** Dorsiflexion gain by gender

	DF Gain			
Gender	Ort.	SS	Median	*p*
Male	10.27	4.73	10	0.37
Female	7.44	6.60	4	

*P:* p value, mean: average, *ss *standard deviation

The mean DF muscle strength gain compared with the contralateral healthy side was 52.28% in males and 30.55% in females.

According to Carayon et al. criteria, 2 patients (10%) achieved excellent results, 10 patients (50%) good, 6 patients (30%) fair, and 2 patients (10%) poor outcomes (Table 3).

**Table 3:** Functional outcomes according to Carayon et al

Results	Sayı	% Değer
Excellent	2	10
Good	10	50
Fair	6	30
Poor	2	10

The Stanmore score indicated excellent results in 6 patients (30%), good in 6 (30%), fair in 4 (20%), and poor in 4 (20%). There was no statistically significant difference in Stanmore scores based on gender or median age (p > 0.05). Patients with excellent Stanmore scores had a significantly shorter median paralysis duration than those with poor or fair results (p < 0.05) (Table 4).

**Table 4:** Stanmore scores by gender, age, and paralysis duration

	Stanmore				
	Excellent	Good	Fair	Poor	
Gender	*n* (%)				*p*
Male	4(66.7)	4(66.7)	1(25.0)	2(50.0)	0.541
Female	2(33.3)	2(33.3)	3(75.0)	2(50.0)	
	**Median**				
Age	31.50	33.00	46.00	38.50	0.989
Duration of paralysis (Months)	7.50	9.00	32.00	54.00	0.002*

*:p<0.05, n: number of samples

The median percentage changes in preoperative and postoperative measurements of the same foot — including the AP tibiotalar angle, calcaneus–first metatarsal angle, lateral talus–first metatarsal angle, lateral talocalcaneal angle, talohorizontal angle, calcaneal pitch angle, talonavicular coverage angle, AP talocalcaneal angle, and AP talus–first metatarsal angle — showed no statistically significant differences according to gender (*p* > 0.05).

Similarly, the percentage changes in the AP tibiotalar angle, calcaneal pitch angle, talonavicular coverage angle, AP talocalcaneal angle, and AP talus–first metatarsal angle were not significantly correlated with patient age (*p* > 0.05).

Postoperatively, the calcaneal pitch angle decreased compared with preoperative measurements in 18 patients, while it increased in 2 patients. Although no radiographic pes planus was observed during follow-up, the limited sample size and follow-up duration prevent definitive conclusions regarding long-term arch integrity. There was a statistically significant correlation between the percentage change in the lateral talocalcaneal angle and duration of paralysis (moderate negative correlation), the percentage change in the lateral talus–first metatarsal angle and duration of paralysis (moderate positive correlation), and the percentage change in the talohorizontal angle and duration of paralysis (strong negative correlation) (*p* < 0.05) (Table 5; Fig. 3). These changes were interpreted as indicating a tendency toward increased hindfoot varus alignment and collapse of the medial longitudinal arch (MLA).

Although the increase in the lateral talocalcaneal angle change with longer paralysis duration suggests a tendency toward hindfoot varus alignment, hindfoot alignment measurements revealed valgus alignment on the unaffected side in 19 patients (95%) and varus in 1 patient (5%). On the affected side, both preoperatively and postoperatively, 17 patients (85%) had valgus alignment and 3 patients (15%) had varus alignment.

**Table 5:** Relationship between Preoperative–Postoperative percentage changes in angles and patients’ Gender, Age, and duration of paralysis

	Male	Female		Age	Duration of paralysis (Months)
Postoperation Same Foot	Median		*p*	*r*; *p*	
AP Tibiotalar Angle Change Percentage	−0.55	2.14	0.070	−0.15; 0.544	−0.07; 0.781
Lateral Talocalcaneal Angle Change Percentage	−0.73	6.91	0.370	−0.14; 0.568	−0.51; 0.023*
Lateral Talus-1st Metatarsal Angle Change Percentage	−23.53	−34.29	0.999	0.072; 0.76	0.554; 0.011*
Calcaneus-1st Metatarsal Angle Change Percentage	2.13	2.74	0.999	0.029; 0.902	−0.262; 0.264
Talohorizontal Angle Change Percentage	19.57	9.85	0.999	−0.084; 0.725	−0.735; <0.001*
Calcaneal Pitch Angle Change Percentage	−14.01	−4.18	0.070	−0.094; 0.695	0.042; 0.860
Talonavicular Overlap Angle Change Percentage	4.00	2.80	0.999	0.160; 0.50	0.302; 0.196
AP Talocalcaneal Angle Change Percentage	0.00	1.75	0.999	0.102; 0.67	0.115; 0.630
AP Talus-First Metatarsal Angle Change Percentage	48.21	43.40	0.999	0.09; 0.71	0.032; 0.895

*:p<0,05

## Discussion

Studies in the literature regarding tibialis posterior tendon transfer have generally focused on surgical technique, the route of transfer, and the fixation point. Tibialis posterior tendon transfer is widely considered the gold standard for the treatment of foot drop. Although there are opinions suggesting that the tibialis posterior tendon transfer provides only a tenodesis effect, most studies have reported that active dorsiflexion (DF) between 15° and 30° is achieved following transfer [8]. A disadvantage of the tibialis posterior muscle as a donor is its limited excursion of approximately 2 cm, whereas the tendons responsible for dorsiflexion normally have an excursion of 3–5 cm [9]. Therefore, careful tension adjustment is required during transfer to achieve sufficient DF. Soares et al. reported using a tendon-to-tendon insertion method and noted that a decrease of about 10° in ankle dorsiflexion could occur from the time of suture application to patient discharge, with a further 5–10° decrease during follow-up. To compensate for this, they recommended securing the tendons while the foot is held in 20° dorsiflexion [10]. In our procedure, we perform the transfer with the ankle held in 10° dorsiflexion under appropriate tension. In cases with long-standing foot drop, Achilles tendon contracture may develop, and Achilles tendon lengthening may be required when passive dorsiflexion is less than 20°. One of the most debated aspects of tibialis posterior tendon transfer is the route used to bring the tendon to the dorsum of the foot. Two common techniques are employed: the interosseous route or the circumtibial route. In the circumtibial route, the tendon is brought subcutaneously around the medial tibial border to reach the dorsal aspect of the foot, providing a longer moment arm but potentially limiting ankle motion. The interosseous route is considered more physiological regarding the direction of tendon movement compared with the circumtibial route. Since the tendon is positioned lateral to the midline on the dorsum of the foot, it also contributes to eversion. However, the major disadvantage of the interosseous route is an increased risk of adhesions, especially if the window in the interosseous membrane is too narrow [11]. There is also a risk of vascular injury with this method. The circumtibial route, on the other hand, provides the tendon with a longer moment arm and thus a mechanical advantage but limits the range of motion. Since the tendon is placed centrally on the dorsum of the foot with this route, it does not contribute to eversion. In one study comparing these two techniques, the interosseous route was shown to provide better dorsiflexion [12].

In our study, since the interosseous route was used and the tendon was passed beneath the extensor retinaculum, inversion problems seen with the circumtibial route and cosmetic issues related to the tendon being palpable subcutaneously were not observed. In tendon-to-tendon transfers, the line of action and insertion point of the tibialis anterior tendon can result in tension that creates not only dorsiflexion but also inversion. Dividing the tibialis posterior tendon and attaching one portion to the tibialis anterior tendon and passing the other portion through the peroneus tertius, extensor digitorum longus, and extensor hallucis longus tendons has been shown to help prevent inversion while also contributing to foot eversion [12, 13]. Although active toe extension is not achieved with this method, including these tendons in the transfer helps prevent foot drop and toe dragging [13].

When fixing the tendon to the bone, whether the fixation point is medial or lateral can lead to varus or valgus deformities. Tension adjustment can be challenging in bone fixations. If the tendon is too short, securing it to the desired point can be difficult [14]. In the study by Rodriguez using the Bridle procedure, a branch of the tibialis posterior tendon passed through the interosseous membrane was transferred to the tibialis anterior tendon and the peroneus longus tendon, which had been rerouted anterior to the lateral malleolus. The other branch was tenodesed directly to the middle cuneiform bone on the dorsum of the foot, providing balanced dorsiflexion while avoiding varus and valgus deformities [15]. However, the impact of tenodesing the tibialis posterior tendon to the middle cuneiform bone on plantarflexion was not addressed in the study. The tibialis posterior tendon plays a supportive role for the medial longitudinal arch (MLA), and its dysfunction can lead to adult-acquired pes planus deformity [16]. However, there are no long-term, comprehensive studies examining the development of pes planus after tendon transfer. In terms of strength parameters, a significant decrease in dorsiflexion strength and approximately one-third reduction in plantarflexion torque compared to the unaffected side have been observed [17].

In a study by Vigasio et al. involving 16 patients who underwent tendon transfer, static foot analysis revealed pes planus in only one patient. Additionally, increased external loading in the metatarsal region and/or forefoot adduction was noted. It is thought that the absence of pes planus after transferring the tibialis posterior tendon to the dorsum of the foot may be due to the counteracting force of the peroneus brevis [18]. Steinau et al. performed pedobarographic analysis in 19 of 53 patients who underwent tendon transfer and found no development of pes planus and a pressure distribution similar to that of the healthy foot. Additionally, compared with the unaffected side, the medial aspect of the midfoot was identified as the region bearing the least load [17]. Recent studies evaluating adult-acquired flatfoot deformity using advanced radiological parameters, such as the work of Colo et al., also highlight the importance of monitoring MLA integrity following procedures that alter the tibialis posterior function [2]. In our radiographic evaluations, no significant changes were observed in AP tibiotalar angle measurements and hindfoot alignment compared with the preoperative state. The statistically significant moderate negative correlation between the percentage change in the lateral talocalcaneal angle and duration of paralysis, moderate positive correlation between the percentage change in the lateral talus–first metatarsal angle and duration of paralysis, and strong negative correlation between the percentage change in the talohorizontal angle and duration of paralysis were interpreted as indicating a tendency toward MLA collapse. Although the collapse of the MLA after tendon transfer was shown to be significantly associated with the duration of paralysis, none of our patients developed pes planus or other deformities. Except for three patients, in whom preoperative varus deformity was already present, no varus feet were observed despite the increase in lateral talocalcaneal angle changes suggesting a tendency toward hindfoot varus alignment. This study has certain limitations. The heterogeneity of etiologies (traumatic, iatrogenic, neurologic) may also affect surgical outcomes, although subgroup analysis was not feasible due to the limited number of patients. Excluding patients with missing radiographs may introduce selection bias. An a priori power analysis could not be performed due to the retrospective study design.

.Data related to tendon transfer were obtained from a single center. The number of patients was small, and the follow-up period was relatively short. The lack of pedobarographic analysis and gait assessment in the preoperative and postoperative periods and the absence of a control group are additional limitations of this study.

## Conclusion

Tibialis posterior tendon transfer using the interosseous membrane route and fixation to the lateral cuneiform bone is an effective option for the surgical management of foot drop. The procedure provides satisfactory restoration of active ankle dorsiflexion, reduces the need for orthotic support, and does not significantly affect foot arch integrity. Optimal surgical technique, proper tensioning, and preoperative strengthening of the tibialis posterior muscle are crucial for successful outcomes.

## Data Availability

Data supporting the findings are available from the corresponding author upon reasonable request.
